# Uptake and 4-week quit rates from an opt-out co-located smoking cessation service delivered alongside community-based low-dose computed tomography screening within the Yorkshire Lung Screening Trial

**DOI:** 10.1183/13993003.01768-2023

**Published:** 2024-04-18

**Authors:** Rachael L. Murray, Panos Alexandris, David Baldwin, Kate Brain, John Britton, Philip A.J. Crosbie, Rhian Gabe, Sarah Lewis, Steve Parrott, Samantha L. Quaife, Hui Zhen Tam, Qi Wu, Rebecca Beeken, Harriet Copeland, Claire Eckert, Neil Hancock, Jason Lindop, Grace McCutchan, Catriona Marshall, Richard D. Neal, Suzanne Rogerson, Harriet D. Quinn Scoggins, Irene Simmonds, Rebecca Thorley, Matthew E. Callister

**Affiliations:** 1 School of Medicine, University of Nottingham, Nottingham, UK; 2 Centre for Prevention, Detection and Diagnosis, Wolfson Institute of Population Health, Queen Mary University of London, London, UK; 3 Department of Respiratory Medicine, Nottingham University Hospitals NHS Trust, Nottingham, UK; 4 Division of Population Medicine, Cardiff University, Cardiff, UK; 5 Division of Infection, Immunity and Respiratory Medicine, Faculty of Biology, Medicine and Health, The University of Manchester, Manchester, UK; 6 Barts Clinical Trials Unit, Centre for Evaluation and Methods, Wolfson Institute of Population Health, Queen Mary University of London, London, UK; 7 York Trials Unit, Department of Health Sciences, University of York, York, UK; 8 Leeds Institute of Health Sciences, University of Leeds, Leeds, UK; 9 Leeds Teaching Hospitals NHS Trust, Leeds, UK; 10 College of Medicine and Health, University of Exeter, Exeter, UK; 11 PRIME Centre Wales, Division of Population Medicine, School of Medicine, Cardiff University, Cardiff, UK

## Abstract

**Background:**

Up to 50% of those attending for low-dose computed tomography screening for lung cancer continue to smoke and co-delivery of smoking cessation services alongside screening may maximise clinical benefit. Here we present data from an opt-out co-located smoking cessation service delivered alongside the Yorkshire Lung Screening Trial (YLST).

**Methods:**

Eligible YLST participants were offered an immediate consultation with a smoking cessation practitioner (SCP) at their screening visit with ongoing smoking cessation support over subsequent weeks.

**Results:**

Of 2150 eligible participants, 1905 (89%) accepted the offer of an SCP consultation during their initial visit, with 1609 (75%) receiving ongoing smoking cessation support over subsequent weeks. Uptake of ongoing support was not associated with age, ethnicity, deprivation or educational level in multivariable analyses, although men were less likely to engage (adjusted OR (OR_adj_) 0.71, 95% CI 0.56–0.89). Uptake was higher in those with higher nicotine dependency, motivation to stop smoking and self-efficacy for quitting. Overall, 323 participants self-reported quitting at 4 weeks (15.0% of the eligible population); 266 were validated by exhaled carbon monoxide (12.4%). Multivariable analyses of eligible smokers suggested 4-week quitting was more likely in men (OR_adj_ 1.43, 95% CI 1.11–1.84), those with higher motivation to quit and previous quit attempts, while those with a stronger smoking habit in terms of cigarettes per day were less likely to quit.

**Conclusions:**

There was high uptake for co-located opt-out smoking cessation support across a wide range of participant demographics. Protected funding for integrated smoking cessation services should be considered to maximise programme equity and benefit.

## Extract

Low-dose computed tomography (LDCT) screening for lung cancer has been shown to reduce lung cancer-specific mortality in two large, randomised trials [1, 2], and implementation of screening is currently underway in many high- and middle-income countries [3]. Of those people responding to an invitation for lung cancer screening and subsequently eligible, between 30% and 50% are currently smoking [4, 5]. Evidence of the impact of screening alone on smoking cessation rates in randomised trials is mixed. Three studies have compared quit rates in participants randomised to LDCT screening *versus* a non-screened control group, with one study showing an increased quit rate in the screened group [6], one study showing no difference between screened and non-screened participants [7], and one study showing a higher quit rate in non-screened participants, although this effect did not persist after intention-to-treat analysis [8]. Importantly, smoking cessation interventions were minimal in all three studies, comprising signposting to existing services, written information to support quit attempts and (in one study) very brief advice [6–8].

## Shareable abstract

**Uptake of stop smoking support co-located within a lung cancer screening programme was high across a range of participant demographics and quit rates were in excess of those reported in other studies at similar time-points without such intervention.** https://bit.ly/3vc8bln

## Graphical abstract

**Figure:**
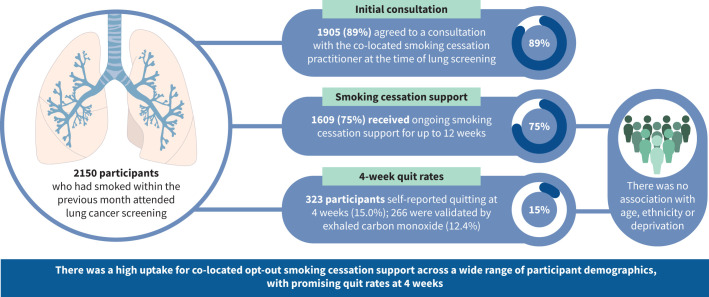
Overview of the study findings.

## Introduction

Low-dose computed tomography (LDCT) screening for lung cancer has been shown to reduce lung cancer-specific mortality in two large, randomised trials [1, 2], and implementation of screening is currently underway in many high- and middle-income countries [3]. Of those people responding to an invitation for lung cancer screening and subsequently eligible, between 30% and 50% are currently smoking [4, 5]. Evidence of the impact of screening alone on smoking cessation rates in randomised trials is mixed. Three studies have compared quit rates in participants randomised to LDCT screening *versus* a non-screened control group, with one study showing an increased quit rate in the screened group [6], one study showing no difference between screened and non-screened participants [7], and one study showing a higher quit rate in non-screened participants, although this effect did not persist after intention-to-treat analysis [8]. Importantly, smoking cessation interventions were minimal in all three studies, comprising signposting to existing services, written information to support quit attempts and (in one study) very brief advice [6–8].

Opt-out delivery of smoking cessation interventions has been proven effective in a number of healthcare settings [9, 10] and thus there is potential to translate this approach to the lung cancer screening setting. Facilitating smoking cessation alongside lung cancer screening has been shown to augment the reduction in lung cancer mortality [11], increase life-years gained [12] and may improve the cost-effectiveness of the screening programme [13]. In addition, there is proven benefit for quitting smoking after a lung cancer diagnosis [14–16]. In September 2022, following a detailed review of efficacy and cost-effectiveness, the UK National Screening Committee recommended that the devolved UK nations implement targeted lung cancer screening for individuals who are high risk due to age and smoking history [17], and in June 2023 a planned national roll-out in England was announced [18]. Recognising the benefits of smoking cessation alongside lung cancer screening, the National Screening Committee recommended integrating smoking cessation services alongside a future screening programme, although no specific implementation details were provided. A recent report on smoking cessation provision within pilot screening programmes being run by the National Health Service (NHS) in England, as part of the Targeted Lung Health Check (TLHC) programme [19], highlighted geographical disparities in availability and relatively low uptake of smoking cessation, which were attributed to a lack of a specified approach and dedicated funding [20]. Systematically targeting those people who smoke and attend a TLHC with appropriately funded stop smoking support has the potential to address these disparities. Many of those attending lung cancer screening typically have long-term tobacco dependence, and it is crucial that effective support strategies are specified and resourced. Determining likely uptake and success is fundamental to assessing the potential utility of this approach. This study, therefore, reports uptake and initial outcomes from a co-located, opt-out smoking cessation support package delivered as part of a community-based lung cancer screening trial in the UK.

## Methods

### Participants and recruitment

Participants in this study were adults who currently smoke attending for a community-based “Lung Health Check” (LHC) including LDCT screening as part of the Yorkshire Lung Screening Trial (YLST) in Leeds, UK [21]. The YLST invitation letter and introductory leaflet were based on those used in the Lung Screen Uptake Trial [22]. YLST was designed as an implementation study for community-based lung cancer screening and thus the initial appropriate material made no reference to the fact that the study was a research project, but instead presented this as a routine NHS service. When eligible participants arrived at the mobile units, the research nature of the study was discussed with participants who then provided written informed consent should they wish to proceed with screening. Of 6819 people who attended their LHC appointment, 17 declined to proceed with screening following further discussion. This approach was approved by the Research Ethics Committee and Confidentiality Advisory Group. No mention was made of smoking cessation services in these materials; the only mention of smoking was the phrase “No judgement on smoking” included in the introductory leaflet. YLST participants were people who had ever-smoked and met any one of three eligibility criteria for screening: the US Preventive Services Task Force 2013 criteria (age 55–80 years, ≥30 pack-years, smoked within 15 years) [23] or a lung cancer risk using the Prostate, Lung, Colorectal and Ovarian Cancer Screening Trial model (PLCO_m2012_) (6-year risk ≥1.51%) [24] or the Liverpool Lung Project (version 2) model (5-year risk ≥5%) [25], as described in detail elsewhere [21].

Screening took place in mobile units located in convenient community-based locations in the vicinity of the invited populations. All consenting individuals who underwent an LDCT scan and who had smoked within the last month or had an exhaled carbon monoxide (CO) reading ≥6 ppm were offered a smoking cessation intervention. This was provided on an opt-out basis by smoking cessation practitioners (SCPs) co-located on the mobile units. Demographic, clinical and behavioural characteristics including baseline smoking data were collected for all YLST participants by the study team. The Index of Multiple Deprivation (IMD) was derived from the participant's home postcode.

### Smoking cessation provision

Unless declined, all eligible individuals attended a consultation with a specialist SCP, trained to National Centre for Smoking Cessation and Training standards [26]. Support was provided in line with National Institute of Health and Care Excellence guidance [27]. This comprised one session of behavioural support delivered face-to-face at the time of the LHC and direct provision of quit aids (either as nicotine replacement therapy (NRT) through delegated prescribing at the visit and/or a commercially available e-cigarette, or a general practitioner prescription arranged for varenicline or bupropion). Any participant willing to meet with the SCP but was unable to do so at the time of the LHC was contacted by telephone within 48 h. Follow-up contact was provided either face-to-face or by telephone, typically weekly but more or less frequently according to participant preference, for up to 4 weeks from the date of the LHC. Where study SCP follow-up was not possible, or if the individual preferred, contact details were passed to the local stop smoking service for referral into community services immediately following the screening visit. All eligible participants were asked for consent to be contacted by telephone at 4 weeks after the screening visit to ascertain smoking status, with optional CO validation if quit (using a Micro+ Smokerlyzer; Bedfont, Maidstone, UK). At this 4-week consultation, informed written consent was collected for participation in a nested substudy, the Yorkshire Enhanced Stop Smoking study (YESS), which tested the effect of adding a personalised smoking cessation intervention to usual stop smoking care [28]. Data presented here relate to engagement with SCPs and quit rates up to and including the 4-week visit prior to activation of the YESS intervention.

### Study duration and the impact of coronavirus disease 2019

YLST opened to recruitment in November 2018 and smoking cessation provision commenced in December 2018. Data presented here relate to outcomes from smoking cessation interventions during the first round of YLST which ran until February 2021. Stop smoking support in YESS was provided for as long as the participant required up to a maximum of 12 weeks from the LHC appointment. Recruitment to YLST was paused between March and June 2020 due to the coronavirus disease 2019 (COVID-19) pandemic. Initially CO validation of self-reported quits was not possible due to lockdown measures for this period and therefore self-reported quits were included in the overall validated quit figures. Following a reduction in lockdown measures in May 2020, CO testing resumed with the study team visiting participants who were not shielding or considered clinically vulnerable to collect CO readings on doorsteps wearing appropriate personal protective equipment. Screening in YLST and face-to-face smoking cessation consultations resumed in July 2020.

### Ethical approval and trial registration

YLST was approved by the Health Research Authority following review by the North West – Greater Manchester West Research Ethics Committee (18/NW/0012) and the Confidentiality Advisory Group (18/CAG/0038). YESS was approved by the Health Research Authority following review by the East Midlands – Derby Research Ethics Committee (18/EM/2019). Both studies are registered with the ISRCTN registry (identifier numbers 42704678 and 63825779, respectively).

### Statistical analysis

All participants were assumed to be still smoking at 4 weeks in the absence of specific information that they had quit [29]. Simple descriptive analyses were undertaken according to whether attendees agreed to ongoing smoking cessation support (Group A), agreed to a consultation with an SCP on the mobile unit but declined ongoing support thereafter (Group B) or declined a consultation with the SCP (Group C). Participants were considered quit if they reported not smoking in the 7 days prior to follow-up (7-day point-prevalent abstinence). The variables selected for hypothesis testing with respect to the outcomes (uptake of cessation support and 4-week quits) were informed by clinical and expert judgement by members of our study team. These factors were investigated using univariate and multivariable logistic regression. The multivariable model was derived by including variables with a p-value <0.20 by univariate analysis and using backwards stepwise selection with a threshold p-value of 0.01 for elimination. Suspected collinearity was checked with a view to simplifying the multivariable model should strong correlation be observed. Trend was investigated by treating ordered variables as continuous in the logistic regression and excluding categories representing when participants “refused” to answer. Odds ratios are presented with 95% confidence intervals. Statistical tests are two-sided. Statistical analyses were conducted using Stata version 17.0 (StataCorp, College Station, TX, USA). A micro-costing approach was used to estimate the mean cost of the intervention and the cost per quit (costs included smoking cessation consultations, pharmacotherapies and e-cigarettes provided by the co-located service).

## Results

**FIGURE 1:**
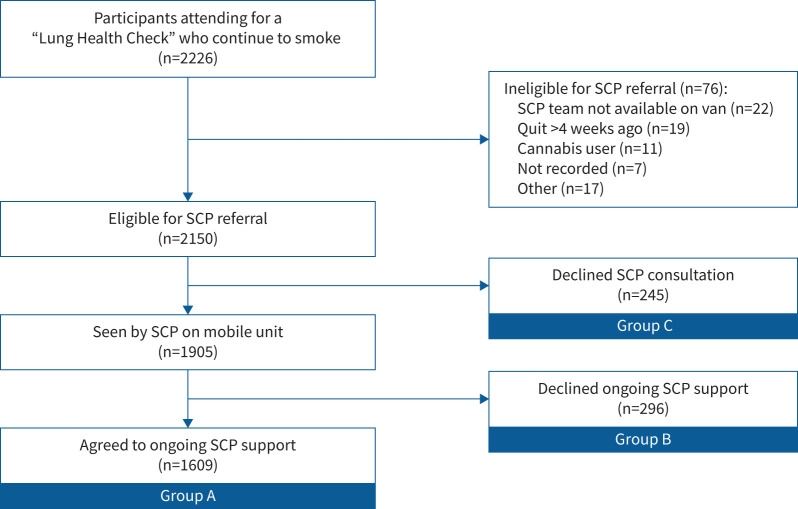
Flow diagram showing outcomes for 2226 participants attending for a “Lung Health Check” who continue to smoke. SCP: smoking cessation practitioner.

Overall 44 943 individuals received a written invitation to telephone risk assessment as described elsewhere [5]. Of these individuals, 22 815 made contact with the telephone triage line for lung cancer risk assessment, and 7853 were found to be eligible for lung cancer screening and did not have any additional exclusion criteria so were offered a LHC appointment. Overall, 1034 people declined the offer or failed to attend their appointment. Of the 6819 individuals attending for LHC, 2226 currently smoked and 2150 were eligible for SCP referral. Of those eligible, 1905 (89%) accepted the referral and were seen by the SCP on the mobile units during the same visit. All participants meeting with the SCP were offered ongoing smoking cessation support, of which 1609 took this up (84% of those meeting the SCP and 75% of those originally eligible for referral). A CONSORT flow diagram, including reasons for ineligibility, is shown in figure 1.

Demographic, clinical and behavioural characteristics of eligible smokers attending the LHC are presented according to uptake of cessation support in table 1. The mean age of the eligible population was 65.5 years, 50% of participants were male and 42% were from the most deprived IMD quintile. Most participants self-identified as White ethnicity (95%) and a majority had left school without qualifications (59%). The mean age of starting smoking was 16 years, median pack-years was 37 and participants most commonly smoked 11–20 cigarettes per day.

**TABLE 1:** Association of baseline factors with uptake of smoking cessation support

	**Total** **eligible** **(Group A+B+C)** **(n=2150)**	**Uptake of support** **(Group A)** **(n=1609)**	**Not taking up support** **(Group B+C)** **(n=541)**	**Univariate** **OR (95% CI)**	**p-value** ^#^
**Age group (years)**					<0.001
<60	488 (22.7)	395 (24.5)	93 (17.2)	1.00	(<0.001)
60–64	542 (25.2)	418 (26.0)	124 (22.9)	0.79 (0.58–1.07)	
65–69	490 (22.8)	355 (22.1)	135 (25.0)	0.62 (0.46–0.84)	
70–74	369 (17.2)	269 (16.7)	100 (18.5)	0.63 (0.46–0.87)	
≥75	261 (12.1)	172 (10.7)	89 (16.5)	0.46 (0.32–0.64)	
**Sex**					0.003
Female	1066 (49.6)	828 (51.5)	238 (44.0)	1.00	
Male	1084 (50.4)	781 (48.5)	303 (56.0)	0.74 (0.61–0.90)	
**IMD quintile**					0.05
1 (most deprived)	904 (42.1)	659 (41.0)	245 (45.3)	1.06 (0.75–1.50)	(0.14)
2	387 (18.0)	285 (17.7)	102 (18.9)	1.10 (0.75–1.62)	
3	340 (15.8)	269 (16.7)	71 (13.1)	1.49 (0.99–2.25)	
4	328 (15.3)	259 (16.1)	69 (12.8)	1.48 (0.98–2.23)	
5 (least deprived)	191 (8.9)	137 (8.5)	54 (10.0)	1.00	
**Ethnicity, self-reported** **^¶^**					0.63
White	2048 (95.3)	1539 (95.6)	509 (94.1)	1.00	
Black	29 (1.4)	21 (1.3)	8 (1.5)	0.87 (0.38–1.97)	
Asian	35 (1.6)	23 (1.4)	12 (2.2)	0.63 (0.31–1.28)	
Other	27 (1.3)	21 (1.3)	6 (1.1)	1.16 (0.46–2.88)	
Prefer not to say^+^	6 (0.3)	4 (0.2)	2 (0.4)		
Missing	5 (0.2)	1 (0.1)	4 (0.7)		
**Education**					0.005
No qualifications	1259 (58.6)	911 (56.6)	348 (64.3)	1.00	(0.003)
O-levels or equivalent	459 (21.4)	360 (22.4)	99 (18.3)	1.39 (1.08–1.79)	
A-levels and above	415 (19.3)	326 (20.3)	89 (16.5)	1.40 (1.07–1.82)	
Prefer not to say^+^	12 (0.6)	11 (0.7)	1 (0.2)		
Missing	5 (0.2)	1 (0.1)	4 (0.7)		
**Cigarettes per day**					0.20
≤10	706 (32.8)	525 (32.6)	181 (33.5)	1.00	(0.20)
11–20	1010 (47.0)	776 (48.2)	234 (43.3)	1.14 (0.91–1.43)	
21–30	230 (10.7)	180 (11.2)	50 (9.2)	1.24 (0.87–1.77)	
>30	65 (3.0)	50 (3.1)	15 (2.8)	1.15 (0.63–2.10)	
Refused	104 (4.8)	70 (4.4)	34 (6.3)	0.71 (0.46–1.11)	
Missing	35 (1.6)	8 (0.5)	27 (5.0)		
**COPD primary care code**					0.12
Yes	550 (25.6)	398 (24.7)	152 (28.1)	0.84 (0.67–1.04)	
No	1600 (74.4)	1211 (75.3)	389 (71.9)	1.00	
**Respiratory symptoms** **^§^**					0.12
Yes	1692 (78.7)	1291 (80.2)	401 (74.1)	1.35 (1.07–1.70)	
No	451 (21.0)	318 (19.8)	133 (24.6)	1.00	
Missing	7 (0.3)	0 (0)	7 (1.3)		
**MRC dyspnoea score**					0.26
0–1	1689 (78.6)	1259 (78.3)	430 (79.5)	1.00	
2–4	454 (21.1)	350 (21.8)	104 (19.2)	1.14 (0.90–1.47)	
Missing	7 (0.3)	0 (0)	7 (1.3)		
**WHO performance status**					
0–1	1910 (88.8)	1440 (89.5)	470 (86.9)	1.00	0.35
2–4	233 (10.8)	169 (10.5)	64 (11.8)	0.86 (0.63–1.17)	
Missing	7 (0.3)	0 (0)	7 (1.3)		
**Perceived personal risk of cancer *versus* other smokers**					<0.001
Lower	242 (11.3)	170 (10.6)	72 (13.3)	1.00	(0.03)
About the same	1397 (65.0)	1074 (66.8)	323 (59.7)	1.40 (1.04–1.90)	
Higher	403 (18.7)	316 (19.6)	87 (16.1)	1.53 (1.06–2.21)	
Refused	74 (3.4)	41 (2.6)	33 (6.1)	0.53 (0.30–0.90)	
Missing	34 (1.6)	8 (0.5)	26 (4.8)		
**Fagerström nicotine dependency**					0.003
<4 (less dependent)	760 (35.4)	556 (34.6)	204 (37.7)	1.00	(0.001)
4–6 (moderately dependent)	905 (42.1)	703 (43.7)	202 (37.3)	1.27 (1.02–1.60)	
7–10 (highly dependent)	258 (12.0)	214 (13.3)	44 (8.1)	1.78 (1.23–2.56)	
Missing	227 (10.6)	136 (8.5)	91 (16.8)		
**Motivation to stop smoking**					<0.001
Don't want to/think I should but don't want to	880 (40.9)	538 (33.4)	342 (63.2)	1.00	(<0.001)
Want to but haven't thought/don't know when	426 (19.8)	376 (23.4)	50 (9.2)	4.78 (3.46–6.61)	
Want to and hope to soon	511 (23.8)	435 (27.0)	76 (14.1)	3.64 (2.75–4.81)	
Really want to and intend to in next 1/3 months	251 (11.7)	226 (14.0)	25 (4.6)	5.75 (3.72–8.88)	
Already quit	24 (1.1)	16 (1.0)	8 (1.5)	1.27 (0.54–3.00)	
Refused	23 (1.1)	10 (0.6)	13 (2.4)	0.49 (0.21–1.13)	
Missing	35 (1.6)	8 (0.5)	27 (5.0)		
**Serious quit attempts in the past year**					<0.001
0	1588 (73.9)	1160 (72.1)	428 (79.1)	1.00	(<0.001)
1	295 (13.7)	241 (15.0)	54 (10.0)	1.65 (1.20–2.26)	
≥2	224 (10.4)	199 (12.4)	25 (4.6)	2.94 (1.91–4.52)	
Refused	7 (0.3)	0 (0.0)	7 (1.3)		
Missing	36 (1.7)	9 (0.6)	27 (5.0)		
**Self-efficacy of quitting**					<0.001
Not at all	424 (19.7)	272 (16.9)	152 (28.1)	1.00	(<0.001)
Slightly/somewhat	663 (30.8)	530 (32.9)	133 (24.6)	2.23 (1.69–2.93)	
Moderately/extremely	979 (45.5)	780 (48.5)	199 (36.8)	2.19 (1.70–2.82)	
Refused	49 (2.3)	19 (1.2)	30 (5.6)	0.35 (0.19–0.65)	
Missing	35 (1.6)	8 (0.5)	27 (5.0)		

Data are presented as n (%), unless otherwise stated. IMD: Index of Multiple Deprivation; MRC: Medical Research Council; WHO: World Health Organization. ^#^: global p-value for non-ordered factors only and global p-value with (p-value for trend) for ordered factors (excluding “refused”); ^¶^: self-reported at eligibility check on mobile van; ^+^: excluded from regression due to small numbers; ^§^: breathlessness, chronic cough, sputum, winter bronchitis, wheeze.

Based on the Fagerström test of nicotine dependency, 35% were less dependent, 42% moderately dependent and 12% highly dependent (data missing for 11% of participants). The majority (74%) of participants had not made a serious quit attempt in the last year, with 14% having made a single attempt and 10% two or more attempts. For those accepting ongoing smoking cessation support, 24% opted for NRT alone, 18% an e-cigarette alone and 33% a combination of both. Pharmacotherapy was selected for 7% of participants, no support was accepted by 14% and data were missing for 4%.

Investigation of individual baseline factors suggested different uptake of ongoing cessation support in participants attending the LHC by age group, sex, education, perceived risk of cancer, nicotine dependency, motivation to stop smoking, quit attempts and self-efficacy of quitting (table 1). When differences in uptake were investigated in a multivariable context, four parameters remained associated with uptake of support (table 2). Specifically, men were significantly less likely to engage in support than women (adjusted OR (OR_adj_) 0.71, 95% CI 0.56–0.89; p=0.002). In addition, those participants with moderate or high nicotine dependency were more likely to engage than those with low nicotine dependency (moderate *versus* low dependency: OR_adj_ 1.43, 95% CI 1.12–1.83; high *versus* low dependency: OR_adj_ 2.25, 95% CI 1.51–3.33; p_trend_<0.001). Greater self-efficacy of quitting predicted higher levels of smoking cessation uptake with evidence of trend (p_trend_<0.001), as did greater motivation to stop smoking (highest motivation in those still smoking *versus* lowest motivation: OR_adj_ 6.36, 95% CI 3.90–10.39; p_trend_<0.001).

**TABLE 2:** Multivariable analyses of factors associated with uptake of cessation support (n=1923)

	**Adjusted OR (95% CI)**	**p-value** ^#^
**Sex**		0.002
Female	1.00	
Male	0.71 (0.56–0.89)	
**Fagerström nicotine dependency**		<0.001
<4 (less dependent)	1.00	(<0.001)
4–6 (moderately dependent)	1.43 (1.12–1.83)	
7–10 (highly dependent)	2.25 (1.51–3.33)	
**Motivation to stop smoking**		<0.001
Don't want to/think I should but don't want to	1.00	(<0.001)
Want to but haven't thought/don't know when	4.66 (3.27–6.65)	
Want to and hope to soon	3.40 (2.51–4.60)	
Really want to and intend to in next 1/3 months	6.36 (3.90–10.39)	
Already quit	1.40 (0.43–4.58)	
Refused	0.56 (0.16–1.94)	
**Self-efficacy of quitting**		<0.001
Not at all	1.00	(0.011)
Slightly/somewhat	1.55 (1.13–2.12)	
Moderately/extremely	1.43 (1.06–1.95)	
Refused	0.36 (0.16–0.81)	

^#^: global p-value for non-ordered factors only and global p-value with (p-value for trend) for ordered factors (excluding “refused”).

Overall 15% of eligible participants (n=323) self-reported quitting at 4 weeks, with 12% (n=266) being CO validated due to COVID-19 social distancing regulations. All but one of the successful quits occurred in the group agreeing to ongoing smoking cessation support where self-reported and validated quit rates were 20% and 17%, respectively. In those agreeing to ongoing support, the mean±sd intervention cost was estimated to be GBP 68.6±44.9 per participant and the corresponding costs per quit were GBP 342.8 (self-reported) and GBP 416.5 (validated), respectively. Considering the whole eligible cohort, the mean±sd intervention cost was GBP 52.4±45.9, with cost per quit at GBP 348.7 (self-reported) and GBP 423.4 (validated).

Baseline characteristics of participants who self-reported a 4-week quit compared with those who did not are shown in table 3. Univariate analyses suggested differences in quitting at 4 weeks by sex, education, cigarettes per day, nicotine dependency, motivation to stop smoking, quit attempts and self-efficacy of quitting (table 3).

**TABLE 3:** Association of baseline factors with 4-week self-reported quitting among eligible smokers (Group A+B+C)

	**Quit by** **4 weeks** **(n=323)**	**Not quitting by** **4 weeks** **(n=1827)**	**Univariate** **OR (95% CI)**	**p-value** ^#^
**Age group (years)**				0.55
<60	77 (23.8)	411 (22.5)	1.00	(0.41)
60–64	90 (27.9)	452 (24.7)	1.06 (0.76–1.48)	
65–69	64 (19.8)	426 (23.3)	0.80 (0.56–1.15)	
70–74	52 (16.1)	317 (17.4)	0.88 (0.60–1.28)	
≥75	40 (12.4)	221 (12.1)	0.97 (0.64–1.46)	
**Sex**				0.03
Female	142 (44.0)	924 (50.6)	1.00	
Male	181 (56.0)	903 (49.4)	1.30 (1.02–1.65)	
**IMD quintile**				0.18
1 (most deprived)	127 (39.3)	777 (42.5)	0.78 (0.51–1.19)	(0.07)
2	48 (14.9)	339 (18.6)	0.68 (0.42–1.09)	
3	61 (18.9)	279 (15.3)	1.05 (0.66–1.67)	
4	54 (16.7)	274 (15.0)	0.94 (0.59–1.51)	
5 (least deprived)	33 (10.2)	158 (8.6)	1.00	
**Ethnicity, self-reported** **^¶^**				0.32
White	312 (96.6)	1736 (95.0)	1.00	
Black	2 (0.6)	27 (1.5)	0.41 (0.10–1.74)	
Asian	6 (1.9)	29 (1.6)	1.15 (0.47–2.80)	
Other	2 (0.6)	25 (1.4)	0.45 (0.10–1.89)	
Prefer not to say^+^	1 (0.3)	5 (0.3)		
Missing	0 (0.0)	5 (0.3)		
**Education**				0.05
No qualifications	169 (52.3)	1090 (59.7)	1.00	(0.02)
O-levels or equivalent	78 (24.1)	381 (20.9)	1.32 (0.99,1.77)	
A-levels and above	73 (22.6)	342 (18.7)	1.38 (1.02–1.86)	
Prefer not to say^+^	3 (0.9)	9 (0.5)		
Missing	0 (0.0)	5 (0.3)		
**Cigarettes per day**				0.002
≤10	140 (43.3)	566 (31.0)	1.00	(0.001)
11–20	127 (39.3)	883 (48.3)	0.58 (0.45–0.76)	
21–30	32 (9.9)	198 (10.8)	0.65 (0.43–0.99)	
>30	8 (2.5)	57 (3.1)	0.57 (0.26–1.22)	
Refused	14 (4.3)	90 (4.9)	0.63 (0.35–1.14)	
Missing	2 (0.6)	33 (1.8)		
**COPD primary care code**				0.10
Yes	71 (22.0)	479 (26.2)	0.79 (0.60–1.05)	
No	252 (78.0)	1348 (73.8)	1.00	
**Respiratory symptoms** **^§^**				0.77
Yes	257 (79.6)	385 (21.1)	1.04 (0.78–1.40)	
No	66 (20.4)	1435 (78.5)	1.00	
Missing	0 (0.0)	7 (0.4)		
**MRC dyspnoea score**				0.27
0–1	262 (81.1)	1427 (78.1)	1.00	
2–4	61 (18.9)	393 (21.5)	0.85 (0.63–1.14)	
Missing	0 (0.0)	7 (0.4)		
**WHO performance status**				
0–1	293 (90.7)	1617 (88.5)	1.00	0.31
2–4	30 (9.3)	203 (11.1)	0.82 (0.55–1.22)	
Missing	0 (0.0)	7 (0.4)		
**Perceived personal risk of cancer *versus* other smokers**				0.25
Lower	44 (13.6)	198 (10.8)	1.00	(0.60)
About the same	206 (63.8)	1191 (65.2)	0.78 (0.54–1.11)	
Higher	64 (19.8)	339 (18.6)	0.85 (0.56–1.30)	
Refused	7 (2.2)	67 (3.7)	0.47 (0.20–1.09)	
Missing	2 (0.6)	32 (1.8)		
**Fagerström nicotine dependency**				0.04
<4 (less dependent)	136 (42.1)	624 (34.2)	1.00	(0.02)
4–6 (moderately dependent)	123 (38.1)	782 (42.8)	0.72 (0.55–0.94)	
7–10 (highly dependent)	35 (10.8)	223 (12.2)	0.72 (0.48–1.08)	
Missing	29 (9.0)	198 (10.8)		
**Motivation to stop smoking**				<0.001
Don't want to/think I should but don't want to	60 (18.6)	820 (44.9)	1.00	(<0.001)
Want to but haven't thought/ don't know when	76 (23.5)	350 (19.2)	2.97 (2.07–4.26)	
Want to and hope to soon	102 (31.6)	409 (22.4)	3.41 (2.43–4.79)	
Really want to and intend to in next 1/3 months	71 (22.0)	180 (9.9)	5.39 (3.69–7.88)	
Already quit	9 (2.8)	15 (0.8)	8.20 (3.45–19.51)	
Refused	3 (0.9)	20 (1.1)		
Missing	2 (0.6)	33 (1.8)		
**Serious quit attempts in the past year**				<0.001
0	187 (57.9)	1401 (76.7)	1.00	(<0.001)
1	80 (24.8)	215 (11.8)	2.79 (2.07–3.76)	
≥2	54 (16.7)	170 (9.3)	2.38 (1.69–3.35)	
Refused	0 (0.0)	7 (0.4)		
Missing	2 (0.6)	34 (1.9)		
**Self-efficacy of quitting**				<0.001
Not at all	38 (11.8)	386 (21.1)	1.00	(<0.001)
Slightly/somewhat	91 (28.2)	572 (31.3)	1.62 (1.08–2.41)	
Moderately/extremely	189 (58.5)	790 (43.2)	2.43 (1.68–3.52)	
Refused	3 (0.9)	46 (2.5)	0.66 (0.20–2.23)	
Missing	2 (0.6)	33 (1.8)		

Data are presented as n (%), unless otherwise stated. IMD: Index of Multiple Deprivation; MRC: Medical Research Council; WHO: World Health Organization. ^#^: global p-value for non-ordered factors only and global p-value with (p-value for trend) for ordered factors (excluding “refused”); ^¶^: self-reported at eligibility check on mobile van; ^+^: excluded from regression due to small numbers; ^§^: breathlessness, chronic cough, sputum, winter bronchitis, wheeze.

When differences in quitting at 4 weeks were investigated in a multivariable context, four parameters remained associated (table 4) indicating that men were more likely to quit than women (OR_adj_ 1.43, 95% CI 1.11–1.84) and individuals still smoking with the highest motivation to quit were more successful in quitting than those with the lowest motivation (OR_adj_ 4.05, 95% CI 2.71–6.05) with strong evidence of trend with level of motivation (p_trend_<0.001). Similarly, those participants who had made serious quit attempts in the past year were more likely to successfully quit compared with those who had not (1 *versus* 0 quit attempts: OR_adj_ 2.00, 95% CI 1.45–2.75), whereas a larger number of cigarettes smoked per day predicted lower quit rate (11–20 *versus* <10 cigarettes per day: OR_adj_ 0.61, 95% CI 0.46–0.80) and evidence of trend with strength of habit (p_trend_<0.001).

**TABLE 4:** Multivariable analyses of factors associated with self-reported quitting at 4 weeks (n=2107)

	**Adjusted OR (95% CI)**	**p-value** ^#^
**Sex**		0.005
Female	1.00	
Male	1.43 (1.11–1.84)	
**Cigarettes per day**		0.01
≤10	1.00	(0.03)
11–20	0.61 (0.46–0.80)	
21–30	0.65 (0.42–1.00)	
>30	0.55 (0.25–1.21)	
Refused	0.66 (0.35–1.24)	
**Motivation to stop smoking**		<0.001
Don't want to/think I should but don't want to	1.00	(<0.001)
Want to but haven't thought/don't know when	2.78 (1.93–4.01)	
Want to and hope to soon	2.99 (2.12–4.24)	
Really want to and intend to in next 1/3 months	4.05 (2.71–6.05)	
Already quit	5.88 (2.33–14.81)	
Refused	2.29 (0.64–8.24)	
**Serious quit attempts in the past year**		0.006
0	1.00	(<0.001)
1	2.00 (1.45–2.75)	
≥2	1.70 (1.18–2.43)	

^#^: global p-value for non-ordered factors only and global p-value with (p-value for trend) for ordered factors (excluding “refused”).

## Discussion

In this study, we report uptake and 4-week quit rates from a co-located, opt-out smoking cessation service delivered in person alongside a community-based LDCT lung cancer screening programme. The offer of cessation support was popular, with 89% of eligible attendees agreeing to an immediate consultation with a SCP and three-quarters accepting longer term support. Around one in seven eligible participants were abstinent from smoking 4 weeks after the LHC, indicating that delivering smoking interventions as an integral part of the screening process is likely to save lives and markedly increase the effectiveness of the screening programme.

The cohort of participants eligible for smoking cessation referral were predominantly from more deprived populations with low levels of educational attainment and the vast majority were of White ethnicity. Most had commenced smoking as teenagers, generally had low motivation to quit smoking and only about one in four eligible participants had made a serious quit attempt in the past year.

In multivariable analyses, having a higher motivation to stop smoking, self-efficacy of quitting and nicotine dependency were associated with greater uptake of smoking cessation support. However, the only demographic or clinical factor which remained associated with the participant engaging with ongoing cessation support was sex, with male participants being about 30% less likely to agree to this than female participants.

Multivariable analyses showed that having a higher motivation to stop smoking and number of quit attempts were associated with self-reported quitting at 4 weeks while a higher number of cigarettes per day was associated with being less likely to quit. In addition, male sex was associated with about a 43% higher likelihood of a self-reported 4-week quit.

While some of these associations are in line with expectations, the differences by sex (both in uptake of support and quit success) are less well documented in the literature. To the best of our knowledge, other studies have not reported engagement with smoking cessation support by sex. However, a secondary analysis of the US National Lung Screening Trial cohort found that female sex was associated with being less likely to attempt quitting [30], in keeping with our results. Differences among male and female smokers participating in lung cancer screening with respect to uptake of smoking cessation support and successful quitting outcomes requires further research.

The finding that people with higher levels of nicotine dependency were more likely to engage with smoking cessation support is somewhat surprising, as previous studies tend to show the reverse effect. This might be due to the opt-out nature of the intervention, coupled with the teachable moment of attendance at lung screening encouraging those people who would not proactively seek quitting support to take advantage of this offer.

Age, deprivation and education level were not significantly associated with uptake of ongoing support and self-reported 4-week quitting in multivariable analyses. The response to invitation to lung cancer screening has been found to be strongly associated with age, sex, ethnicity, deprivation as well as smoking status itself [5], which is of concern as those most at risk of lung cancer seem less willing or able to engage with screening overall. However, the data presented here are encouraging, suggesting co-delivery of smoking cessation support alongside LDCT screening is acceptable across a wide range of participants. A complete process evaluation of the provision of stop smoking support in this setting, including the acceptability of the opt-out offer at the time of lung screening, has been undertaken and results will be published separately.

### Comparison with other literature

Direct comparison with other studies is limited by differences in participant populations, follow-up duration and definitions of quitting for calculating quit rates. Several studies report quit data at 12 weeks following the screening visit, so are not comparable to the data presented here collected only 4 weeks after screening [31–34]. The only study documenting quit rates at a similar time-point was an analysis of people undergoing LDCT screening in the UK Lung Screening Pilot. This documented 75 people of 758 screened (9.9%) self-reporting a quit 2 weeks after receiving their screening results (so likely at a similar time-point to our 4-week time-point) following provision of standard smoking cessation advice leaflets and signposting to existing services [6]. Our self-reported quit rate of 15% at 4 weeks following the screening visit is thus higher than that reported with no bespoke smoking cessation service. Additionally, the cost per self-reported quit reported in this study (GBP 342) was lower than the findings from Stop Smoking Services England, where the 12-week cost per self-reported quit was GBP 404 in 2015–2016 (equivalent to GBP 419 when adjusted to 2019/2020 prices) [35, 36].

### Strengths and limitations

With 2150 eligible participants attending for lung cancer screening and continuing to smoke, this study is the largest to report uptake and outcomes from an opt-out face-to-face smoking cessation service co-located with a community-based mobile screening unit. Furthermore, the comprehensive analysis of demographic, clinical and behavioural parameters allows a thorough characterisation of the factors associated with uptake of smoking cessation support and 4-week quit rate. Limitations of the data include the fact that this intervention took place within a research study, which might limit generalisability due to the possibility of the health volunteer effect. However, as discussed in the Methods section, YLST was designed as an implementation study for lung cancer screening and as such no mention of research was made in the initial invitation material nor the telephone call during which eligibility was assessed. Research was mentioned for the first time when eligible participants attended the mobile screening units; only 0.25% of attendees declined to participate in the study following discussion on the mobile unit. Thus, although this intervention did take place within a research study, the results maybe applicable to a more general population due to these considerations. Another limitation of the data presented here is the lack of quit information beyond 4 weeks; most comparable studies report quit outcomes at 3 and 12 months, thus precluding direct comparison. However, the focus of this article is on factors influencing the uptake of stop smoking support in this setting to inform future policy discussions as to how best to integrate smoking cessation intervention within lung screening programmes. Further research will report longer term outcomes in this population, as well as uptake and effectiveness of smoking cessation support at future (incident) rounds of LDCT screening. The study population is predominantly White, which while reflective of the lung cancer screening eligible population in the UK, limits the generalisability of the findings to other ethnic groups.

### Summary

Smoking cessation interventions have the potential to augment the clinical benefit of lung cancer screening programmes by increasing the life-years gained by the programme through facilitating successful quit attempts in participants. Recognising this, the UK National Screening Committee has recommended integrating smoking cessation alongside a future national screening programme. Uptake of smoking cessation support reported here was high, with 89% of eligible people agreeing to a face-to-face consultation with an SCP and 75% accepting ongoing support. Furthermore, results suggest that this service is well received across a broad range of service-users. Four-week self-reported quit rates of 15% are in excess of quit rates at similar time-points following screening without such interventions. Policymakers must consider ring-fenced funding for bespoke smoking cessations services to run alongside LDCT screening programmes to realise their full potential in improving lung cancer outcomes, and in doing so reduce all-cause smoking-related morbidity and mortality.

## Shareable PDF

10.1183/13993003.01768-2023.Shareable1 This one-page PDF can be shared freely online.
Shareable PDF ERJ-01768-2023.Shareable
