# Supplementary material for: Uptake and 4-week quit rates from an opt-out co-located smoking cessation service delivered alongside community-based low-dose computed tomography screening within the Yorkshire Lung Screening Trial
Source: Eur Respir J. 2024 Apr 18;63(4):2301768. doi: 10.1183/13993003.01768-2023 (PMC11024392; doi:10.1183/13993003.01768-2023)
Supplement: Supplementary file 1 [file ERJ-01768-2023.Shareable.pdf]

EUROPEAN RESPIRATORY JOURNAL
ORIGINAL RESEARCH ARTICLE
R.L. MURRAY ET AL.

SHAREABLE PDF

Uptake and 4-week quit rates from an opt-out co-located smoking cessation service delivered alongside community-based low-dose computed tomography screening within the Yorkshire Lung Screening Trial

Rachael L. Murray, Panos Alexandris, David Baldwin, Kate Brain, John Britton, Philip A.J. Crosbie, Rhian Gabe, Sarah Lewis, Steve Parrott, Samantha L. Quaife, Hui Zhen Tam, Qi Wu, Rebecca Beeken, Harriet Copeland, Claire Eckert, Neil Hancock, Jason Lindop, Grace McCutchan, Catriona Marshall, Richard D. Neal, Suzanne Rogerson, Harriet D. Quinn Scoggins, Irene Simmonds, Rebecca Thorley and Matthew E. Callister

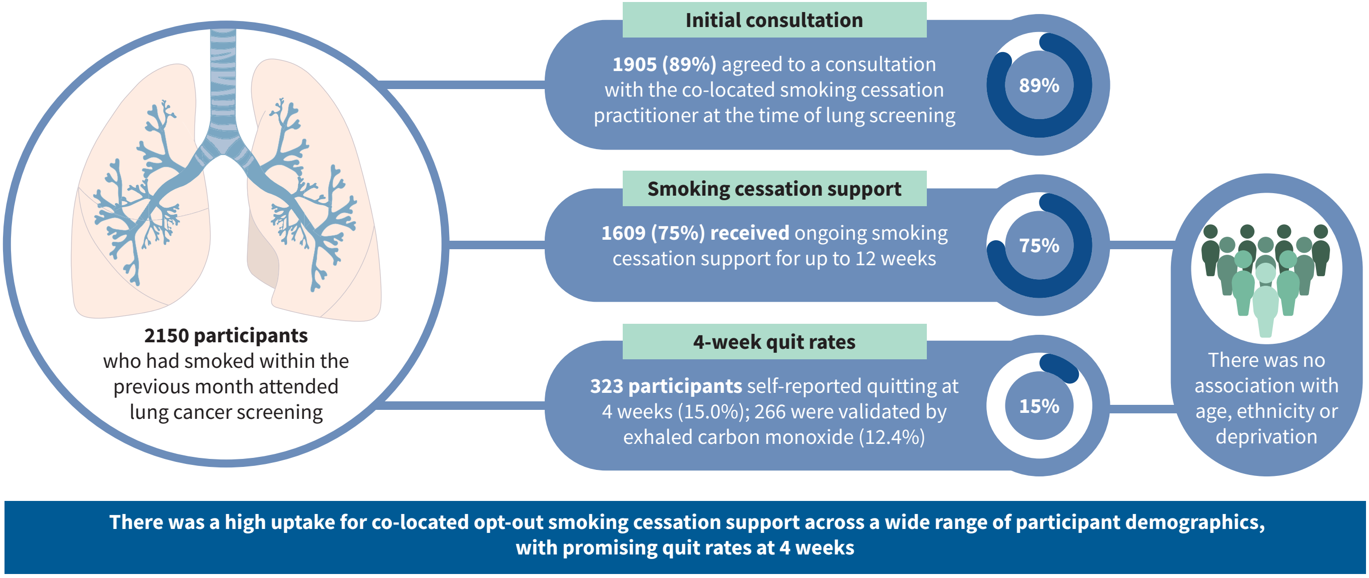

GRAPHICAL ABSTRACT Overview of the study findings.

EUROPEAN RESPIRATORY JOURNAL
ORIGINAL RESEARCH ARTICLE
R.L. MURRAY ET AL.

SHAREABLE PDF

Uptake and 4-week quit rates from an opt-out co-located smoking cessation service delivered alongside community-based low-dose computed tomography screening within the Yorkshire Lung Screening Trial

Rachael L. Murray[1], Panos Alexandris[2], David Baldwin[3], Kate Brain[4], John Britton[1], Philip A.J. Crosbie[5], Rhian Gabe[6], Sarah Lewis[1], Steve Parrott[7], Samantha L. Quaife[2], Hui Zhen Tam[6], Qi Wu[7], Rebecca Beeken[8], Harriet Copeland[8], Claire Eckert[8], Neil Hancock[8], Jason Lindop[9], Grace McCutchan[4], Catriona Marshall[8], Richard D. Neal[10], Suzanne Rogerson[9], Harriet D. Quinn Scoggins[11], Irene Simmonds[8], Rebecca Thorley[1] and Matthew E. Callister[8,9]

[1]School of Medicine, University of Nottingham, Nottingham, UK. [2]Centre for Prevention, Detection and Diagnosis, Wolfson Institute of Population Health, Queen Mary University of London, London, UK. [3]Department of Respiratory Medicine, Nottingham University Hospitals NHS Trust, Nottingham, UK. [4]Division of Population Medicine, Cardiff University, Cardiff, UK. [5]Division of Infection, Immunity and Respiratory Medicine, Faculty of Biology, Medicine and Health, The University of Manchester, Manchester, UK. [6]Barts Clinical Trials Unit, Centre for Evaluation and Methods, Wolfson Institute of Population Health, Queen Mary University of London, London, UK. [7]York Trials Unit, Department of Health Sciences, University of York, York, UK. [8]Leeds Institute of Health Sciences, University of Leeds, Leeds, UK. [9]Leeds Teaching Hospitals NHS Trust, Leeds, UK. [10]College of Medicine and Health, University of Exeter, Exeter, UK. [11]PRIME Centre Wales, Division of Population Medicine, School of Medicine, Cardiff University, Cardiff, UK.

Corresponding author: Rachael L. Murray (rachael.murray@nottingham.ac.uk)

Check for updates

Shareable abstract (@ERSpublications)
Uptake of stop smoking support co-located within a lung cancer screening programme was high across a range of participant demographics and quit rates were in excess of those reported in other studies at similar time-points without such intervention. https://bit.ly/3vc8bln

Cite this article as: Murray RL, Alexandris P, Baldwin D, *et al*. Uptake and 4-week quit rates from an opt-out co-located smoking cessation service delivered alongside community-based low-dose computed tomography screening within the Yorkshire Lung Screening Trial. *Eur Respir J* 2024; 63: 2301768 [DOI: 10.1183/13993003.01768-2023].

This extracted version can be shared freely online.

Copyright ©The authors 2024.

This version is distributed under the terms of the Creative Commons Attribution Licence 4.0.

This article has an editorial commentary: https://doi.org/10.1183/13993003.00550-2024

Received: 16 Oct 2023
Accepted: 1 March 2024

Abstract

Background Up to 50% of those attending for low-dose computed tomography screening for lung cancer continue to smoke and co-delivery of smoking cessation services alongside screening may maximise clinical benefit. Here we present data from an opt-out co-located smoking cessation service delivered alongside the Yorkshire Lung Screening Trial (YLST).

Methods Eligible YLST participants were offered an immediate consultation with a smoking cessation practitioner (SCP) at their screening visit with ongoing smoking cessation support over subsequent weeks.

Results Of 2150 eligible participants, 1905 (89%) accepted the offer of an SCP consultation during their initial visit, with 1609 (75%) receiving ongoing smoking cessation support over subsequent weeks. Uptake of ongoing support was not associated with age, ethnicity, deprivation or educational level in multivariable analyses, although men were less likely to engage (adjusted OR (OR_{adj}) 0.71, 95% CI 0.56–0.89). Uptake was higher in those with higher nicotine dependency, motivation to stop smoking and self-efficacy for quitting. Overall, 323 participants self-reported quitting at 4 weeks (15.0% of the eligible population); 266 were validated by exhaled carbon monoxide (12.4%). Multivariable analyses of eligible smokers suggested 4-week quitting was more likely in men (OR_{adj} 1.43, 95% CI 1.11–1.84), those with higher motivation to quit and previous quit attempts, while those with a stronger smoking habit in terms of cigarettes per day were less likely to quit.

Conclusions There was high uptake for co-located opt-out smoking cessation support across a wide range of participant demographics. Protected funding for integrated smoking cessation services should be considered to maximise programme equity and benefit.
